# Evolution of the CDKN1C-KCNQ1 imprinted domain

**DOI:** 10.1186/1471-2148-8-163

**Published:** 2008-05-29

**Authors:** Eleanor I Ager, Andrew J Pask, Helen M Gehring, Geoff Shaw, Marilyn B Renfree

**Affiliations:** 1 Department of Zoology, The University of Melbourne, Melbourne, Victoria, 3010, Australia

## Abstract

**Background:**

Genomic imprinting occurs in both marsupial and eutherian mammals. The *CDKN1C *and *IGF2 *genes are both imprinted and syntenic in the mouse and human, but in marsupials only *IGF2 *is imprinted. This study examines the evolution of features that, in eutherians, regulate *CDKN1C *imprinting.

**Results:**

Despite the absence of imprinting, CDKN1C protein was present in the tammar wallaby placenta. Genomic analysis of the tammar region confirmed that *CDKN1C *is syntenic with *IGF2*. However, there are fewer LTR and DNA elements in the region and in intron 9 of *KCNQ1*. In addition there are fewer LINEs in the tammar compared with human and mouse. While the CpG island in intron 10 of *KCNQ1 *and promoter elements could not be detected, the antisense transcript *KCNQ1OT1 *that regulates *CDKN1C *imprinting in human and mouse is still expressed.

**Conclusion:**

CDKN1C has a conserved function, likely antagonistic to IGF2, in the mammalian placenta that preceded its acquisition of imprinting. CDKN1C resides in synteny with IGF2, demonstrating that imprinting of the two genes did not occur concurrently to balance maternal and paternal influences on the growth of the placenta. The expression of *KCNQ1OT1 *in the absence of CDKN1C imprinting suggests that antisense transcription at this locus preceded imprinting of this domain. These findings demonstrate the stepwise accumulation of control mechanisms within imprinted domains and show that *CDKN1C *imprinting cannot be due to its synteny with *IGF2 *or with its placental expression in mammals.

## Background

Eutherians and marsupials (therian mammals) diverged between 125–145 million years ago [1-3]. Genomic imprinting, in which the monoallelic expression of certain genes depends on the parent of origin, occurs in both therian mammal lineages. This is in contrast to all other vertebrate species including monotreme mammals in which imprinting of endogenous genes has not been demonstrated [4-7]. Monoallelic expression negates the advantage of diploidy, namely the masking of deleterious alleles. The cost of imprinting must, therefore, be outweighed by its potential benefits to the genetic fitness of the individual. The parental conflict hypothesis proposes that imprinting is the product of asymmetric selection on parental genomes, with selection favouring the expression of paternal genes that increase the amount of maternal nutrient transfer while expression from maternally-inherited genes will be favoured if they reduce nutritional demands on the mother [8-10]. The placentas of marsupials and eutherians mediate the transfer of nutrients between mother and young and the placentas of both groups of mammal express imprinted genes [11-16]. However, the marsupial placenta is comparatively short-lived and the majority of support for growth and development of the marsupial young occurs during an extended and complex period of lactation [17]. Thus marsupials are ideal models in which to examine the evolution of imprinting and its association with mammalian placentation.

*CDKN1C *(expressed from the maternally inherited allele) and *IGF2 *(expressed from the paternally inherited allele) are syntenic, and implicated in growth disorders such as Beckwith-Wiedemann syndrome [18]. *IGF2 *is paternally expressed in mouse and human [19] and stimulates cell cycle progression. IGF2 is highly conserved in all vertebrates and is expressed during marsupial placentation, as it in the eutherian placenta [14]. *IGF2 *is also imprinted in the tammar placenta, indicating imprinting at this locus evolved before the eutherian-marsupial split [14]. In contrast, progression through the cell cycle is negatively controlled by cyclin-dependent kinase (CDK) inhibitors, such as p57^KIP2^, encoded by the gene *CDKN1C *[20-22]. *CDKN1C *is expressed from the maternal allele in many tissues of mouse and human, including the placenta [23,24], but is bi-alellically expressed (ie, not imprinted) in the tammar wallaby [11]. Placental development is disrupted in mice carrying a maternally-derived mutation in *Cdkn1c *[18,25] and mutations in *CDKN1C *are associated with trophoblastic disease in humans [26,27]. Unlike *IGF2*, *CDKN1C *is rapidly evolving in mammals and has low homology between mouse, human, and tammar, with only the CDK-inhibiting domain conserved [14,28]. Divergence in the structure of p57^KIP2 ^suggests functional specialisation in different species and may explain species-specific patterns of expression, subtle differences in the pathologies of human and mouse *CDKN1C *mutants [29], and the absence of imprinting of this gene in the tammar [14].

Most imprinted genes aggregate within the genome and in each imprinted domain gene order and imprint status show considerable similarity between mouse and human [30-32]. *IGF2 *and *CDKN1C *are syntenic, and reside in an imprinted cluster on mouse distal chromosome 7. Included in this region are the maternally expressed genes *Ipl/Tssc3*, *Slc22a1l/Tssc4*, *Mash2/Ascl2*, and *Cdkn1c *and the paternally expressed genes *Igf2 *and *Ins2*. Gene order and the imprint status of most genes within this region are conserved with human chromosome 11p15.5 [33]. The entire region is regulated by two differentially methylated imprinting control regions (ICRs) one of which is the *KCNQ1OT1 *antisense-RNA transcript, that regulates the expression of *Cdkn1c*, *Kcnq1*, and *Ascl2 *[31,32]. Conserved synteny may reflect the co-ordinated transcriptional regulation of several imprinted genes within each domain [34,35]. The *IGF2*-*CDKN1C *region is highly conserved even between human and chicken, despite a lack of imprinting in the chicken [36]. Nevertheless, synteny may facilitate the co-evolution of maternal and paternal imprints in adjacent regions of the genome [35,37], possibly by the spread of imprinting mechanisms from one locus to another, as described by the bystander hypothesis [38]. *INS *and *IGF2 *are syntenous in mouse, human and tammar [15,39,40]. Extension of this synteny from *IGF2 *to *CDKN1C *has recently been described in marsupials [40]. This is of particular importance given that *IGF2 *and *INS *are imprinted in the tammar, as in mouse and human, but *CDKN1C *is not [14,15].

Differentially methylated regions (DMRs), often found at CpG islands in or near imprinted genes, clearly contribute to the regulation of imprinted expression in eutherians [34,41-43]. However, not all imprinted genes depend on this mechanism. Antisense transcripts and various forms of chromatin modification also regulate imprinted expression in eutherians [44-46]. *Kcnq1ot1 *(*LIT1*) is a paternally expressed antisense transcript originating from intron 10 of the maternally expressed *Kcnq1 *(*KvLQT1*) gene [47,48]. The human *KCNQ1OT1 *promoter starts approximately 40 bp upstream of the transcription initiation site and spans 300 to 350 bp [49], although associated transcription factor binding sites may extend as far as 2000 bp upstream [36,47]. The transcription initiation site is located in a CpG island that is methylated in oocytes, but not in sperm. Methylation blocks transcription of the maternal allele of *Kcnq1ot1 *and deletion of either the CpG island or *Kcnq1ot1 *increases expression of *Cdkn1c*, *Kcnq1*, and *Ascl2*, indicating its role in the coordinated regulation of these loci [48].

DNA methylation as a regulator of imprinted expression may have evolved from the molecular systems associated with the silencing of transposable elements. The host defence hypothesis suggests that DNA methylation fortuitously provided a mechanism to regulate parental specific gene expression and similar circumstances may have led to the silencing function of antisense transcripts at imprinted loci [50,51]. Indeed, many imprinted genes are found in association with repeat sequences such as non-LTR elements (long and short interspersed transposable elements – LINEs/SINEs), DNA elements, and endogenous retroviruses [37,52-54] and are all known to attract methylation [34].

We determined the expression of *CDKN1C *and the cellular localisation of its protein, p57^KIP2^, in the placenta of the tammar wallaby because of their evolutionary divergence from eutherian mammals. We examined whether *CDKN1C *became imprinted after it acquired a role in placental development, or if its placental expression pre-dated the evolution of its imprinting. In addition, to gain insight into the evolution of imprinting within this cluster, the structure and repeat distribution of the *CDKN1C *domain, including *KCNQ1 *and the ICR, *KCNQ1OT1*, were investigated in the tammar.

## Results

### P57^KIP2 ^localises to the tammar placenta

**Figure 1:**
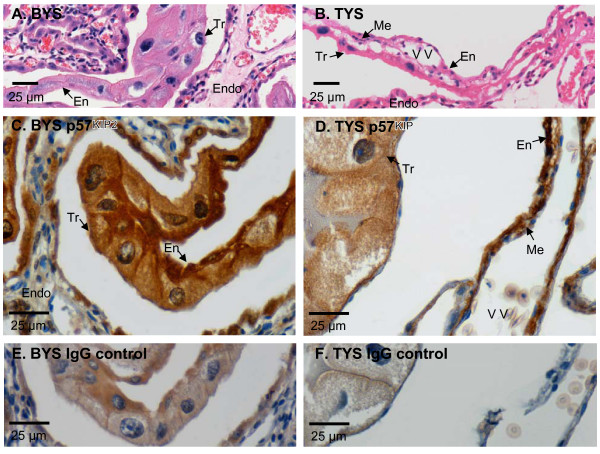
**Immunohistochemical localisation of p57^KIP2 ^in the tammar yolk sac.** Haematoxylin and eosin stained bilaminar yolk sac (BYS, A.) and trilaminar yolk sac (TYS, B.) showing trophoblast cells (Tr), yolk sac endodermal cells (En), and, in the trilaminar yolk sac only, mesenchymal cells (Me) and vitelline vessels (VV). The trophoblast lies adjacent to the maternal endometrium (Endo). p57^KIP2 ^was found in the cytoplasm and nucleus of most trophoblast and endodermal cells in the bilaminar (C.) and trilaminar (D.) yolk sac placenta. Although mesenchymal cells were also stained, fewer were positive. IgG antibody controls also showed weak cytoplasmic, but not nuclear staining (bilaminar, E; trilaminar, F). Day 25 of gestation stages are shown, but staining did not change notably over the stages examined (Days 19 to 26).

While *CDKN1C *is imprinted in eutherians and vital for placentation, it is not imprinted in the tammar [11]. The chorio-vitelline placenta of the tammar (*Macropus eugenii*) consists of two functional regions, a vascular and non-vascular one. The vascular, trilaminar placenta is thought to be the primary site of gas exchange, while that of the avascular, bilaminar region, the predominant site for nutritional exchange [55,56]. We used immunohistochemistry to examine P57^KIP2 ^in both regions of the marsupial placenta. P57^KIP2 ^was localised in the cytoplasm and nucleus of all cell types in the bilaminar and trilaminar yolk sac placenta (Figure 1). However, while the protein was consistently present in many cells of the yolk sac endoderm and trophoblast, staining in the mesenchymal and endothelial cells was sporadic (Figure 1). No notable difference in staining intensity or in the cross-reactivity of different cell types was evident for the stages examined (days 19–26 of the 26.5 day gestation). Weak non-specific cytoplasmic staining was evident in the yolk sac endoderm and, to a lesser extent, the trophoblast in matched goat IgG treated controls (Figure 1), but was clearly distinguishable from the dense staining in both the cytoplasm and nucleus with p57^Kip2 ^Ab-7. There was no cytoplasmic or nuclear staining in no-antibody negative controls (not shown).

### CDKN1C expression increases in the final days of gestation

**Figure 2:**
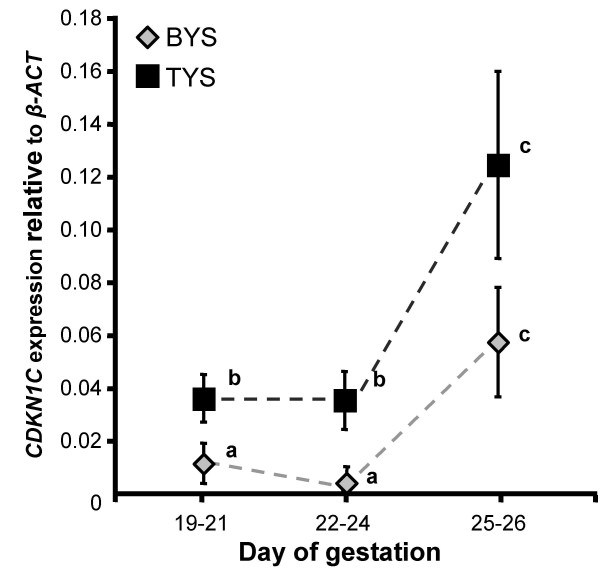
***CDKN1C* expression in the bilaminar (BYS-diamonds) and trilaminar (TYS-squares) yolk sac placenta. **Stages examined; 19–21 (n = 8), 22–24 (n = 7), and 25–26 (n = 6). *CDKN1C *mRNA levels were higher in the TYS between days 19 and 24 than the BYS, but most notable was the significant increase in expression, in both regions of the yolk sac, after day 24 (days 25–26). Means sharing the same superscripted letters are not significantly different (P > 0.05). Means with different superscripts are significantly different (P ≤ 0.05).

*CDKN1C *was expressed in the tammar placenta, but immunohistochemistry did not indicate if there were changes in the quantity of protein over time. We used quantitative RT-PCR to examine quantitative changes in the expression of *CDKN1C*. Between days 19 and 24, *CDKN1C *expression was higher in the trilaminar yolk sac than in the bilaminar yolk sac placenta (Bonferroni adjusted paired t-test, n ≥ 5, α ≤ 0.039) (Figure 2). However, in the two days before birth (days 25 and 26) there was no longer a significant difference in *CDKN1C *expression between vascular and avascular regions (Bonferroni adjusted paired t-test, n = 5, α = 0.158), but there was a significant increase in *CDKN1C *expression in both regions of the placenta (Bonferroni adjusted unpaired t-test, n ≥ 5, α ≤ 0.043).

### Genomic analysis – gene order is conserved in the tammar and eutherians

**Figure 3:**
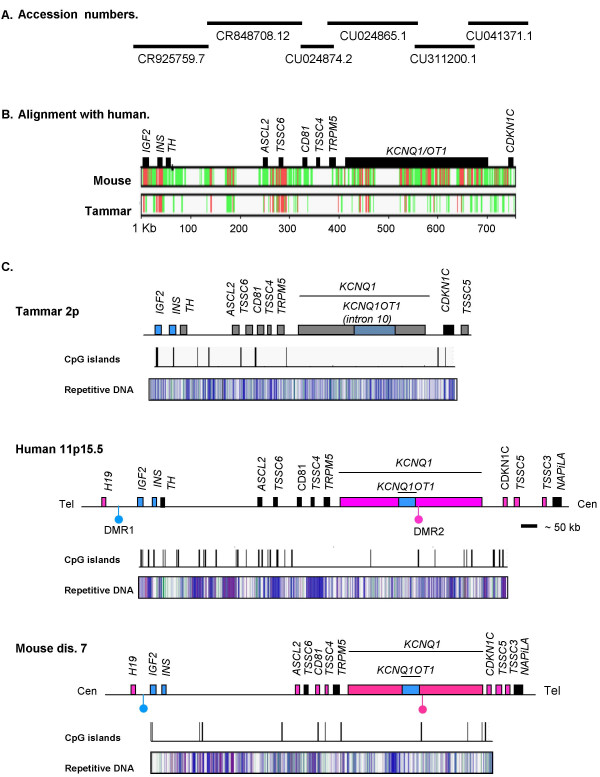
**Structure of the IGF2 to CDKN1C region.** Tammar BAC clones from GenBank (NCBI) were used to derive the tammar sequence (A). A multiple PIP alignment of mouse and tammar sequence against human (B). Conserved regions (red) and regions of homology (green) occur mostly in gene-rich regions (black boxes), but also in the intergenic regions. The *KCNQ1OT1 *region is highly conserved between mouse and human, but divergent in tammar (indicated by white). *IGF2 *to *CDKN1C *in the tammar (C, top), human (C, middle), and mouse (C, bottom). In human and mouse the region includes two imprinted domains regulated the paternally methylated DMR1 and the maternally methylated DMR2 (blue and pink lollipops respectively). Mouse and human regions have been modified from published figures [31, 33, 74, 75]. Gene order is conserved between human, mouse, and tammar. The imprint status of eight of the fourteen genes are conserved between mouse and human (paternally expressed, blue; maternally expressed, pink; biallelic, black). In the tammar, *IGF2 *and *INS *are also paternally expressed, while *CDKN1C *is biallelic. The imprint status of the remaining genes has not been determined (grey). CpG islands and the distribution of repetitive elements are similar between mouse and human and tammar. However, the CpG island associated with DMR2 is absent in the tammar. There are similar numbers of LINE/SINE elements (blue bars) and simple repeats (green) in all species, but fewer DNA elements and LTR elements (pink and purple bars respectively) in the tammar.

We examined the region containing *IGF2 *and *CDKN1C *in the tammar and compared this to human and mouse. A BLAST-N with tammar *CDKN1C *sequence identified a single clone of tammar genomic DNA (GenBank: CU041371.1, clone MEKBa-36303). Successive BLAST-N searches identified five more overlapping clones joining the *IGF2*-contianing clone to the *CDKN1C*-containing clone [Genbank: CR925759.7, Genbank: CR848708.12, Genbank: CU024874.2, Genbank: CU024865.1, Genbank: CU311200.1] (Figure 3A). Each set of overlapping clones showed 100% identity over at least 1000 bp. The overlapping clones provided almost 1 Mb of continuous sequence spanning the domains bordered by *IGF2 *and *CDKN1C*.

Several regions were conserved or homologous between human, mouse, and tammar as indicated by a multi-species percent identity plot (PIP) (Figure 3B). Most of the conserved regions corresponded to genomic locations with a high gene density, such as the gene cluster bordered by *ASCL2 *and *TRPM5*. However, the large intergenic region between *TH *and *ASCL2 *also had two regions conserved or homologous in all three species (see *Genomic Analysis *below).

A PIP of the orthologous region of human against the tammar sequence was generated. Regions of high homology to human indicated the location of putative exons in the tammar sequence. The sequence of each possible exon was entered into a BLASTN to confirm gene identity and was aligned back to human sequences to confirm the location of exon-intron boundaries (data not shown). Of the nine genes located between *IGF2 *and *CDKN1C *in human, eight were identified in the tammar with *KCNQ1OT1 *the only gene not found by homology (Figure 3C). Exons homologous to human *TSSC5*, which lies downstream of *CDKN1C*, were also identified, but the partial sequence available did not extend to cover the entire gene.

**Figure 4:**
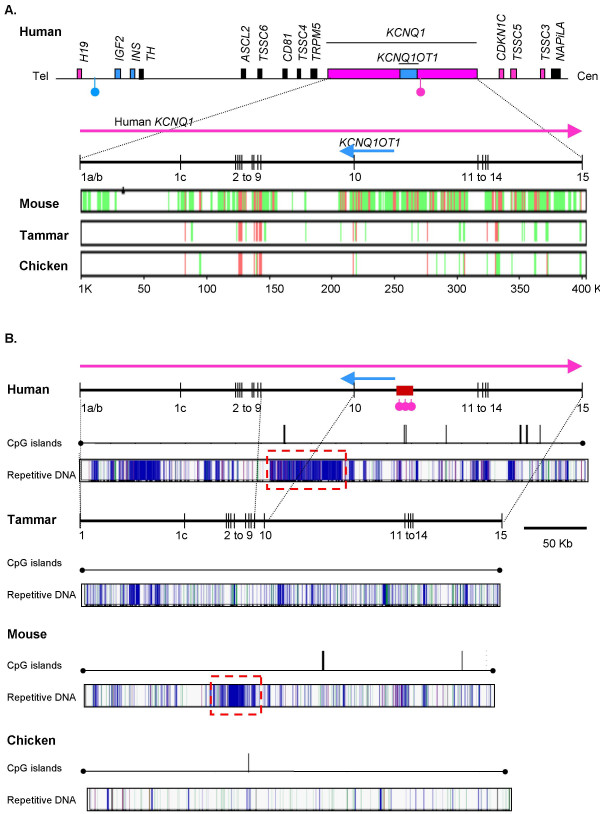
**The *KCNQ1* region in human, tammar, mouse and chicken.** Exons 1a/b to 15 are shown as vertical lines and intronic distances by horizontal lines. The *KCNQ1OT1 *transcript (blue line) is transcribed from a promoter within the maternally methylated CpG island (red box with pink lollipops). A multiple PIP alignment of mouse, tammar and chicken against human *KCNQ1 *(A.). Conserved (red) and homologous regions (green) are common between mouse and human, especially in intron 10 (spanning the majority of *KCNQ1OT1*). Both tammar and chicken are divergent from human (divergence indicated by white) in this, and other intronic regions, with only exons showing high homology. The exon-intron structure of human and tammar is highly conserved, with the notable exception of intron 9, which is markedly reduced in the tammar (B). Human, mouse and chicken have at least one CpG island in the *KCNQ1 *region, while tammar has no CpG islands. There is a notable reduction in the number of repetitive elements in chicken compared to the mammalian species. DNA elements (pink), LTR elements (purple), simple repeats (green) and non-LTR (LINE/SINEs, blue). A conserved region of highly repetitive sequence in mouse and human in intron 9 is indicated by a red dashed box.

**Figure 5:**
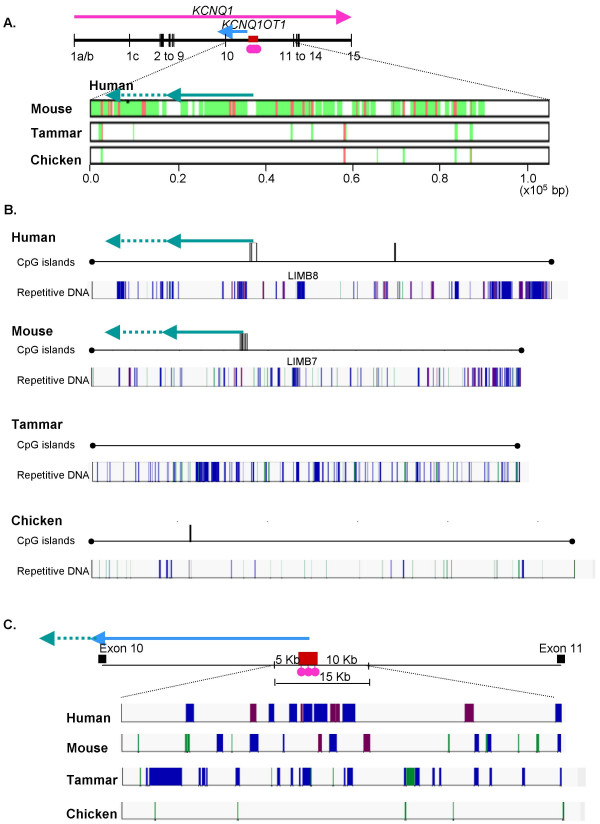
**A multiple PIP alignment of mouse, tammar, and chicken intron 10 against human (A).** There are many areas of high conservation (red) and homology (green) between human and mouse, but little with tammar and chicken. The transcription start site (TSS) of *KCNQ1OT1 *is indicated by a blue arrow. Although the TSS is not highly conserved between mouse and human, the upstream promoter region is, as is the position of the CpG island relative to the TSS (B.). Although mouse and human have a similar numbers and types of repetitive elements, only the L1MB element upstream of the TSS may be conserved. In tammar, as in other regions, there are fewer DNA elements (pink) and LTR elements (purple) compared to human and mouse. Simple repeats (green) and non-LTR (LINE/SINEs, blue) are similar in human, mouse and tammar, but significantly fewer in chicken.

Gene order was highly conserved between human, mouse, and tammar (Figure 3C), as was gene structure. A multi-species PIP of the *KCNQ1 *gene of human against mouse, tammar, and chicken, shows conserved gene structure in all four species, with regions of homology corresponding to exon locations (Figure 4A). However, while the exon-intron structure of *KCNQ1 *was generally highly conserved, intron 4 was absent in chicken and intron 9 was significantly reduced in length in the tammar and in chicken (Figure 4B). In mouse and human the *KCNQ1OT1 *transcript starts in intron 10 and extends into intron 9. Mouse intron 9 and 10 of *KCNQ1 *had significant homology with human introns 9 and 10, while no homology was evident for the tammar or chicken (Figure 5A).

### Genomic analysis – Tammar sequences lack the KCNQ1OT1 promoter and CpG island

The CpG island in intron 10 of *KCNQ1 *is essential for imprinted expression of the *KCNQ1OT1 *transcript in mouse and human. We examined the CpG content of the orthologous region in the tammar. There were 24 CpG islands, grouped into nine clusters, in the sequence spanning *IGF2 *to *CDKN1C *in the tammar, while in human there were 51 (in 31 clusters) and in mouse 29 (in 12 clusters, Figure 3C). Six CpG islands in the human sequence were greater than 1000 bp in length with the longest island 2671 bp. In comparison, only one of the islands in the tammar sequence was longer than 1000 bp (1373 bp). However, mouse also had only two CpG islands over 1000 bp (the longest reaching 1025 bp). Although both human and mouse had fewer CpG islands in *KCNQ1 *compared to the remaining sequence assessed (see *IGF2*-*CDKN1C *in Figure 3C), there were no CpG islands in *KCNQ1 *of the tammar (Figure 4B). Like human and mouse, chicken had a CpG island in *KCNQ1 *(Figure 4B). Despite differences in the CpG island content of *KCNQ1 *in the human and tammar, the overall percent GC was similar (50.9% in the tammar and 51.4% in human).

In human, mouse and chicken at least one CpG island was located in intron 10 of *KCNQ1 *(Figure 5B). In human and mouse the position of the CpG island and the *KCNQ1OT1 *promoter region were highly conserved (Figure 5A and 5B). Although a CpG island was also present in the chicken intron 10, it is not clear if this is orthologous, as no significant homology to the *KCNQ1OT1 *transcription start site could be found, and the CpG island was located approximately 20 and 15 Kb downstream of the orthologous CpG islands in human and mouse respectively.

### Expression analysis of KCNQ1O1

**Figure 6:**
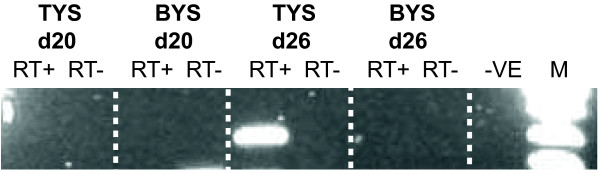
**Expression analysis of the *KCNQ1OT1*.** Primers designed from intron 10 of *KCNQ1 *were used to determine expression of the *KCNQ1OT1 *anti-sense RNA. Primers yield a single 400 bp band as confirmed by genomic DNA PCR (result not shown). Expression was only detected in the trilaminar portion of the placenta (TYS) and not the bilaminar (BYS) and only in the final stages of pregnancy. RT+ denotes samples that have been reverse transcribed. RT- denotes DNased RNA, also used in the PCR reaction to ensure no DNA carryover. -VE represents the negative control reaction in which template was omitted and M, indicates DNA marker.

Primers were designed within the tammar *KCNQ1 *intron 10 to determine if it still encoded a *KCNQ1OT1 *antisense RNA molecule despite its lack of conservation with human and mouse. Since primers did not span an intron, extracted RNA was DNased and an aliquot removed for PCR to ensure there was no genomic DNA contamination (RT- control). Surprisingly, transcription of the putative *KCNQ1O1 *gene was detected in the trilaminar, but not the bilaminar placenta and only during the final stages of pregnancy (Figure 6). The resulting PCR band was sequence verified to ensure amplification of the correct product.

### Genomic analysis – Analysis of repeat distribution in the IGF2-CDKN1C region

Repeat sequences may contribute to the evolution and or regulation of many imprinted regions and so the distribution of repetitive elements in the tammar *IGF2-CDKNIC *region was assessed. Two regions of high homology were identified in the intergenic DNA between *TH *and *ASCL2 *(Figure 3B) and represent areas of high LINE/SINE density in all three species (Figure 3C).

**Figure 7:**
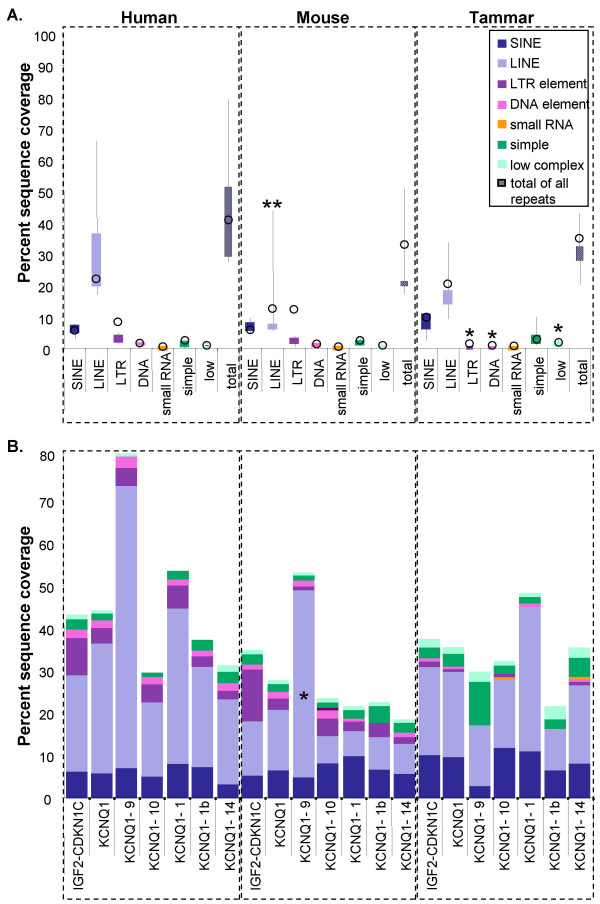
**The sequence coverage of repetitive elements in sequences from human, mouse, and tammar.** A box plot showing the percent of total sequence masked by SINEs (dark blue), LINEs (light blue), LTR elements (purple), DNA elements (pink), simple repeats (teal), and low complexity regions (pale green) in intron 1, 1b, 9, 10, and 14 of *KCNQ1 *(A). The percent sequence coverage for each element over the entire region from *IGF2-CDKN1C *is shown by open circles. There was significantly less sequence masked by LTR and DNA elements in tammar compared to both mouse and human, while there was significantly more sequence occupied by low complexity regions (*). There was significantly less sequence occupied by LINEs in mouse compared to human (**). There was a large range in the sequence covered by LINEs in all species, but particularly in human and mouse. The percent sequence occupied by different types of repetitive elements in the region from *IGF2-CDKN1C*, in all of *KCNQ1*, and in introns 9, 10, 1, 1b, and 14 assessed separately (B). The relative percentage of sequence occupied by LINEs is noticeably more than the percentage occupied by LINEs in any other intron from KCNQ1 and this increase was significant for mouse (*). No other significant differences in the relative amounts of sequence covered by different types of elements was seen between regions within species. However, there was also a noticeable increase in the relative amount of simple repeats in intron 9 of tammar compared to other *KCNQ1 *introns in the tammar.

The percent sequence covered by all repetitive elements in the region from *IGF2-CDKN1C *was not significantly different between species (Figure 7A). When the KCNQ1 region was assessed separately, the percent covered by all repetitive sequences in introns 1, 1b, 9, 10, and 14 (the largest introns) still did not differ significantly between species. However, the percentage of sequenced covered by specific classes of repetitive sequence did differ significantly between species (Figure 7A).

There were significantly fewer long-terminal repeat (LTR) elements (GLM; α = 0.000) and DNA elements (GLM; α = 0.001) in tammar *KCNQ1 *introns 1, 1b, 9, 10, and 14 compared to the same introns in mouse and human, while there were significantly more low complexity regions (α = 0.000). However, the lower percentage of sequence covered by LTR and DNA elements in the tammar compared to mouse and human was also evident across the entire region from *IGF2 *to *CDKN1C *(Figure 3C, 4B, and 7A). However, the relative proportion of LINE/SINEs and simple repeats was not notably different between species in either KCNQ1 or the entire IGF2-CDKN1C region (Figure 3C and 7A).

In both human and mouse there was high concentration of repetitive elements in intron 9, largely due to an increase in LINEs, which are largely absent in tammar intron 9 (Figure 4B and 7B). This was significantly different in mouse compared to tammar (Grubb's critical = 1.71; n = 5; z value = 1.785), but not with human (Grubb's critical = 1.71; n = 5; z value = 1.646). A decrease in SINEs is associated with imprinted regions [56]. However, there was no significant difference in the percentage of sequence covered by SINEs between tammar and human and mouse.

Despite similarities in the overall proportions of different types of repetitive elements in mouse and human, no specific repetitive elements in intron 9, intron 10, or at the transcription start site, were clearly identified as homologous (Figure 5B). However, in both human and mouse an L1MB element (L1MB8 in human and L1MB7 in mouse) was located approximately 10 to 15 Kb upstream of the *KCNQ1OT1 *transcription start site (Figure 5B).

## Discussion

We have confirmed the synteny of *CDKN1C *with *IGF2 *in the tammar wallaby and shown it is highly conserved between the human, mouse and tammar. IGF2 and p57^KIP2 ^antagonistically regulate growth of the eutherian placenta. Although *CDKN1C *is not imprinted in the tammar wallaby, it is expressed in the placenta and so could antagonise the growth promoting effects of IGF2 on the marsupial placenta. Furthermore, the antisense transcript, *KCNQ1OT1*, known to regulate *CDKN1C *imprinting in eutherians is also expressed in the marsupial placenta. However, a CpG island and promoter, orthologous to eutherian *KCNQ1OT1*, were absent in the tammar genome. These data suggest that imprinting of the *CDKN1C *gene is not contingent on its synteny with IGF2, expression in the placenta or the expression of the *KCNQ1OT1 *gene.

The p57^KIP2 ^protein was present in the trophoblast, yolk sac endoderm, and some mesenchymal cells of the yolk sac placenta. In eutherians, P57^KIP2 ^binds cyclin-dependent kinases in the nucleus. In the tammar, P57^KIP2 ^was found in the nucleus as well as the cytoplasm of cells in all tissue types, suggesting that this protein is functional in the marsupial placenta, as in eutherians. However, members of the KIP family, including p57^KIP2^, also have roles outside their CDK activity that may account for their cytoplasmic location [57-60].

*CDKN1C *mRNA expression was higher in the trilaminar placenta compared to the bilaminar, but this difference was only significant when expression was low (days 22 to 24). More conspicuous was the significant increase in *CDKN1C *expression in both regions of the yolk sac placenta in the two days before birth (days 25 to 26). The vascular region of the placenta develops rapidly between days 19–24 to facilitate transfer of nutrients to the developing fetus, consistent with a low *CDKN1C *expression. The increase in *CDKN1C *expression immediately before birth is consistent with retarded growth of the placenta at this time [54]. The slightly higher expression of *CDKN1C *in the trilaminar placenta may represent the terminal differentiation of haematopoietic tissue in this region that requires exit from the cell cycle. *CDKN1C *expression in the marsupial placenta is therefore consistent with a role for P57^KIP2 ^in the inhibition of cell cycle progression and regulation of marsupial placental growth.

*IGF2 *and *CDKN1C *are co-expressed in the placentas of human, mouse, and tammar, suggesting these genes were also co-expressed in the placenta of the therian ancestor. The antagonistic functional relationship between these genes is also likely to have existed in the ancestor of marsupials and eutherians. However, the *CDKN1C *imprint must have been acquired later and only in the eutherian lineage [14].

All genes located between *IGF2 *and *CDKN1C *in human were also syntenic in the tammar. However, homology to *KCNQ1OT1 *was lacking. Despite high conservation of *KCNQT1 *exons between mouse, human and tammar, intron 10 of the tammar *KCNQT1 *gene showed no homology to the *KCNQ1OT1 *eutherian transcript. The promoter region and associated CpG island, conserved in human and mouse [49], could not be detected in the tammar. Intron 9 of *KCNQ1*, which encodes the terminal end of *KCNQ1OT1*, also lacks homology with its eutherian counterpart and is considerably shorter in the tammar (and chicken) than in human and mouse. Since *KCNQ1OT *is a non-coding RNA transcript, its sequence conservation with eutherians is not critical to its function [48]. We therefore examined the expression of *KCNQT1 *intron 10 by RT-PCR. Despite the sequence divergence, transcription was detected suggesting that an antisense RNA *KCNQ1OT1*-orthologue exists in marsupials as in eutherians. This is especially interesting, since *CDKN1C *is not imprinted in marsupials, despite the presence of *KCNQ1OT1*. It will be important to determine whether *KCNQ1 *and *KCNQ1OT1 *are imprinted in the tammar, as in eutherians. As none of the promoter elements of eutherian *KCNQ1OT1 *were identified, it is possible that marsupial *KCNQ1OT1 *is regulated by a completely different means and has not yet been co-opted into regulating imprinting of *CDKN1C*. However, it does show that evolution of *KCNQ1OT1 *preceded imprinting of *CDKN1C*.

Throughout the rest of the *IGF2*-*CDKN1C *region, there was little cross-species sequence homology in the intergenic regions. However, two regions of high homology were identified in mammals between the *TH *and *ASCL2 *genes. Both sites of homology corresponded to regions of high LINE/SINE density suggesting conservation of repetitive elements may account for the sequence homology, rather than any regulatory or control regions. These repeats may be important for establishing a boundary region between two independently regulated domains, leading to their conservation.

There were significantly fewer DNA elements and endogenous retroviruses (LTR elements) across the entire region, spanning both the imprinted *IGF2 *domain and the non-imprinted *CDKN1C *domain. However, in both mouse and human there was a considerable increase in repetitive elements in intron 9, when compared with the tammar. This was due to the accumulation of LINEs that appear to coincide with the termination of the *KCNQ1OT1 *transcript. These results suggest that the truncation of *KCNQ1 *intron 9 in the tammar and the increase in LINEs in mouse and human may be associated with the evolution of *KCNQ1 *imprinting in the region.

## Conclusion

Despite its lack of imprinting, marsupial *CDKN1C *is expressed in the developing placenta where it may antagonise the actions of IGF2 on cell-cycle progression, as it does in eutherians. Since *CDKN1C *resides in synteny with *IGF2*, imprinting of the two genes did not occur concurrently to balance maternal and paternal influences on the growth of the placenta. The expression of *KCNQ1OT1 *in the absence of CDKN1C imprinting suggests that antisense transcription at this locus may have preceded imprinting of this domain. These findings demonstrate the stepwise accumulation of control mechanisms within imprinted domains and show that *CDKN1C *imprinting is not due to its synteny with *IGF2 *or its placental expression in mammals.

## Methods

### Animals

Adult females carrying fetuses in the final third of gestation (day 19 to day 26 of a 26.5 day gestation) [61] were euthanised either by cervical dislocation or by an anaesthetic overdose (sodium pentobarbitone, 60 mg/ml, to effect) and portions of the bilaminar (BYS) and trilaminar (TYS) yolk sac placenta collected as previously described [55,62]. All experiments were approved by the University of Melbourne Animal Experimentation Ethics Committees and the animal handling and husbandry were in accordance with the CSIRO/Australian Bureau of Agriculture and National Health and Medical Research Council of Australia (1990) guidelines.

### Immunohistochemistry

Small pieces of uterus with placenta attached were collected from days 19–21, 22–24, and 25–26 of pregnancy and fixed in 4% PFA before paraffin embedding. After sectioning at 7 μm, dewaxing and rehydration, an antigen retrieval step of 90°C for 60 min in 10 mM sodium citrate buffer pH 6.0 was performed. A rabbit polyclonal p57^Kip2 ^Ab-7 antibody (NeoMarkers, *RB-1637*-P0) was used to localise p57^KIP2 ^in the yolk sac. The antibody epitope corresponded to the C-terminus, which is conserved in marsupial and eutherian p57^KIP2 ^proteins [14]. Sections were blocked with 10% normal goat serum/TBS/1% BSA for 25 minutes at room temperature and subsequently incubated overnight at 4°C with the primary antibody (0.004 g/L). IgG antibody (Santa Cruz, normal rabbit IgG, # *sc-2027*) negative controls (0.004 g/L) and no-antibody (diluent only) negative controls were run concurrently with the p57^Kip2 ^Ab-7 antibody. A biotinylated secondary antibody, goat anti-rabbit (DAKO, # *E0432*), was used with ABComplex/HRP kit (DAKO, # *K0355*) and colour developed with DAB Chromagen tablets (DAKO, # *S3000*). Sections were counterstained in haematoxylin. Sections from each individual were treated in at least two independent immunohistochemical runs to assess the consistency of staining.

### RT-PCR

Approximately 300 ng of total RNA (GenElute Mammalian Total RNA Kit, Sigma, # *RTN70*) was DNase treated (DNA-free, Ambion, # 1906) and an aliquot removed (designated the RT- control) prior to an oligo (dT)_12–18_primed cDNA synthesis reaction (SuperScript First Strand Synthesis System for RT-PCR, Invitrogen, # *11904-018*) (designated RT+ reactions). PCRs, with primers designed within intron 10 of the *KCNQ1 *gene (Fwd 5'-TTCTGCTGGTTCAGCATCAC; Rev 5'-GATGGGAGGGAAGGACATTT;

PCR conditions consisted of: 95°C for 2 mins, followed by 40 cycles of: 95°C for 30 s, 60°C for 30 s, 72°C for 1 min), were performed on RT- and RT+ samples to control for the complete absence of contaminating genomic DNA carryover. Resulting products were sequenced to verified correct product amplification.

### Quantitative RT-PCR

Approximately 300 ng of DNase treated (DNA-free, Ambion, # 1906) total RNA (GenElute Mammalian Total RNA Kit, Sigma, # *RTN70*) was used in an Oligo (dT)_12–18 _primed cDNA synthesis reaction (SuperScript First Strand Synthesis System for RT-PCR, Invitrogen, # *11904-018*). SYBR green (Quantitect, # *204143*) was used in a quantitative PCR on the MJ Research Opticon 2. Primer sequences and PCR conditions are given in Table 1. Melting curve analysis and agarose gel electrophoresis ensured a single product. *B-ACT *was used as a calibrator and endogenous gene control (forward primer 5' GATCCATTGGAGGGCAAGTCT 3' and reverse primer 5' CCAAGATCCAACTACGAGCTTTTT 3'). Standard curves confirmed linearity over three orders of magnitude of yolk sac cDNA dilutions. Reactions were performed in triplicate and the data exported into Microsoft Excel and Systat for analysis. The standard curve showed linearity over three orders of magnitude of yolk sac cDNA dilutions, indicating that the primers work over a range of cDNA concentrations and had a correlation co-efficient of 0.999. The standard deviation of C_T _values amongst triplicates ranged from 0.01 to 0.99 indicating that within each triplicate C_T _values were within 1 cycle of each other.

**Table 1:** Quantitative RT-PCR primer sequences and reaction conditions for *CDKN1C*.

Primers	
Fw primer (5'to 3')	GCCTCAAACCCTTTCACCT
Rv primer (5'to 3')	CGCTTACGGGTCCTCTGAT

**Reagents**	

MasterMix	10 ul, 2×
Primer mix	150 uM final each
H_2_O	4.1 ul
cDNA	5 ul, ~5 ng

**Conditions**	

	50°C 10 min
	95°C 15 min

39 ×	95°C 30 sec
	60°C 20 sec
	72°C 40 sec
	78°C 1 sec
	Plate read

	72°C 1 min
Melting curve	55 – 90°C, 0.5°C, 1 sec
	72°C 5 min
	15°C Hold

Melting curve analyses were performed after each PCR and one sample from each triplicate was assessed by gel electrophoresis to confirm there was no contamination.

### Genomic analysis

A partial cDNA sequence of tammar *CDKN1C *(Suzuki et al., 2005) was used in a BLAST-N (NCBI; [63] to identify a BAC clone of tammar genomic DNA. Overlapping clones were identified by successive BLAST-Ns of the terminal 1000 – 3000 bp of each new clone. All human, mouse, and chicken sequences were obtained from Ensembl [64]. Sequences were examined for CpG islands (EMBOSS CpG Plot; [65]). CpG islands were searched using default settings (200 bp with a CG percentage greater than 50% for a 10 window set and an observed over expected of 0.6).

Repetitive elements were searched using species-specific databases available as option settings in RepeatMasker [66] and Censor [67,68]. However, *Monodelphis domestica *was used in analysis of *Macropus eugenii *when performing a Censor search and, for RepeatFinder, the Mammalian repeat database was searched as no specific marsupial repeat databases were available. Repeat sequences were assessed in the region from *IGF2 *to *CDKN1C *as a single unit and in all large introns of *KCNQ1 *(introns 1, 1b, 9, 10, and 14), each as single units. Only large introns were assessed as smaller introns have fewer repetitive elements of all types and, as such, comparisons between introns 9 and 10 where assessed against similarly sized introns.

Percent identity plots (PIPMaker and MultiPIPMaker: [69] were used to identify conserved and homologous regions between species and to locate putative exons in the tammar. BLASTN (NCBI tools) and ClustalW (EBI tools; [70]) were used to confirm the identity of genes/exons. *KCNQ1OT1 *(intron 10 of *KCNQ1*) was assessed with mVISTA [71] to identify sequence homology within the putative promoter region. The percent sequence conservation, which refers to number of sequences with 70% homology over at least 100 bp.

### Statistical analyses

Univariate statistics (means, variation) and Grubbs' test for outliers were performed using Microsoft Excel (version Microsoft XP 2003). Analysis of quantitative gene expression followed standard procedures [72,73]. Paired (BYS versus TYS) and unpaired (comparisons between stages) t-tests were Bonferroni adjusted (using Systat Version 10.2) to correct for multiple comparisons. The percent coverage of different types of repetitive elements was arcsine transformed and differences between species assessed by general liner model (GLM) least squares analyses using Systat Version 10.2. Grubbs' test for outliers was used to assess if the percent sequence coverage of each element was significantly different in one intron compared to all other similarly sized introns. Quantitative data are presented as means ± s.e.m. for quantitative RT-PCR or as box plots or stacked column graphs for repeat sequence data. Statistical significance was at the 5% level (α-value less than 0.05).

## List of abbreviations

BYS: bilaminar yolk sac placenta; TYS: trilaminar yolk sac placenta; *CDKN1C*: cyclin dependent kinase inhibitor 1C; *IGF2*: insulin-like growth factor 2; *INS*: insulin; *KCNQ1*: Potassium channel, voltage-gated, KQT-like subfamily, member 1; *KCNQ1OT1*: *KCNQ1 *overlapping transcript 1; LINE: long interspersed element; LTR: long terminal repeat; SINE: short interspersed element.

## Authors' contributions

MBR, GS, AJP, EIA, HMG and other members of the Renfree Research Group collected the samples; EIA and HMG performed all the experiments and genome analysis. All authors provided suggestions for the experiments, and read, modified and approved the final manuscript.
